# Ultra Wideband Radar Cross Section Reduction by Using Polarization Conversion Metasurfaces

**DOI:** 10.1038/s41598-018-36542-6

**Published:** 2019-01-24

**Authors:** Edris Ameri, Seyed Hassan Esmaeli, Seyed Hassan Sedighy

**Affiliations:** 1 School of Electrical Engineering, Iran University of Science and Technology, Tehran, Iran; 2 School of New Technologies, Iran University of Science and Technology, Tehran, Iran

## Abstract

In this paper, a polarization conversion metasurface (PCM) is designed for ultra wideband radar cross section (RCS) reduction. The proposed polarization conversion metasurface consists of double-heads arrow unit cell with its 90°, 180° and 270° rotated ones to create the destructive interferences cancellation and radar cross section (RCS) reduction, consequently. The proposed metasurface demonstrates ultra-wide band 10-dB RCS reduction from 9 to 40 GHz (126.5%) for normally TM- and TE- polarized incident waves. The good agreement between the simulation and measurement results at 0°, 20° and 40° incident angles prove the idea, also. The ultra wideband RCS reduction of the proposed metasurface as well as its low profile, light weight and low cost prove its high capability compared with the state of the art references.

## Introduction

Broadband RCS reduction has many applications in stealth military platforms such as unmanned aerial vehicles (UAVs), aircrafts and missiles. Different conventional methods have been proposed to reduce RCS such as using radar absorbent materials (RAMs) and shaping. RAMs which usually need thin lossy sheet thickness convert the electromagnetic energy (EM) to heat with frequency and incident angle dependence behavior. Also, the shaping method is based on redirecting the scattered waves from the target by changing its geometrical shape which has some practical drawbacks such as changing the target aerodynamic properties^1^.

Recently, a new method has been proposed in^2,3^ based on wave redirecting concept by combination of artificial magnetic conductors (AMC) and perfect electric conductors (PEC) tiles in chessboard like configuration. In this method, while the PEC tiles reflect the wave out-phase, the AMC tiles behave as perfect magnetic conductor (PMC) and reflect the wave in-phase in limited bandwidth which create destructive interferences cancellation and RCS reduction, consequently. To increase the RCS reduction bandwidth, two different AMC unit cells have been used instead of AMC-PEC configuration in^4–6^. In these structures, the phase difference of the reflected wave from the AMC unit cells is 180° ± 37° in wide bandwidth which results in more than 10-dB RCS reduction. For example, two different AMC unit cells have been proposed in^4^ results in 85% 10-dB RCS reduction bandwidth. In^6^, a topology optimization algorithm was employed to design two different AMC unit cells with 95% RCS reduction bandwidth by using two layers structure. Using more than two AMC unit cells and special arrangements can be employed to enhance the RCS reduction bandwidth as discussed in^7–10^. Recently, authors proposed two and three layers AMC metasurface with 108% and 109% RCS reduction, respectively^11,12^.

Another method of RCS reduction is using of polarization conversion metasurfaces (PCM) that convert the incident waves to its cross-polarized ones which can employ to scattered waves cancellation^13–15^. A 3-bit coding metasurface has been introduced in^16^ for RCS reduction across wide frequency bandwidth. A low RCS metasurface has been designed based on the optimization algorithm and far-field scattering pattern analysis where the RCS reduction achieved from 7 GHz to 14 GHz^17^. A one-dimensional metasurface based on size-adjustable meta-atoms with ultra-wideband specular reflection reduction has been designed in^18^, also. These structures have been used in terahertz, also^19^. The double arrows unit cell has been used in^13^ to perform polarization conversion metasurface in wide bandwidth, 85%. The polarization conversion realizations in terahertz regime have been considered in^20–23^, also.

In this paper, a thin, low cost, light weight, and ultra wideband polarization conversion metasurface is designed to achieve ultra wideband RCS reduction. In this structure, the double-heads arrow unit cell array and its mirrors create 180° phase difference at both *x* and *y* directions which results in scattered wave cancellation and RCS reduction in normal directions for ultra-wide bandwidth from 9 to 40 GHz, 126.5%. The proposed structure is implemented on very thin and low cost commercially available FR-4 substrate placed on top of a ground plane with air gap. Based on the best author knowledge, the RCS reduction bandwidth of the proposed metasurface is significantly higher than the early published references such as^11,12^. In addition to this ultra wideband RCS reduction bandwidth, the proposed metasurface has very light weight which is due to using ultra thin FR-4 substrate with 0.25 mm thickness and air gap. It should be mentioned that RCS reduction surface weight and cost have important roles in its practical applications. Moreover, the 10-dB RCS reduction bandwidth of the proposed surface is more than 63% for TE polarization and more than 32% for TM ones up to 50° incident angles.

## Design Methodology

### PCM Unit cell Design

**Figure 1:**
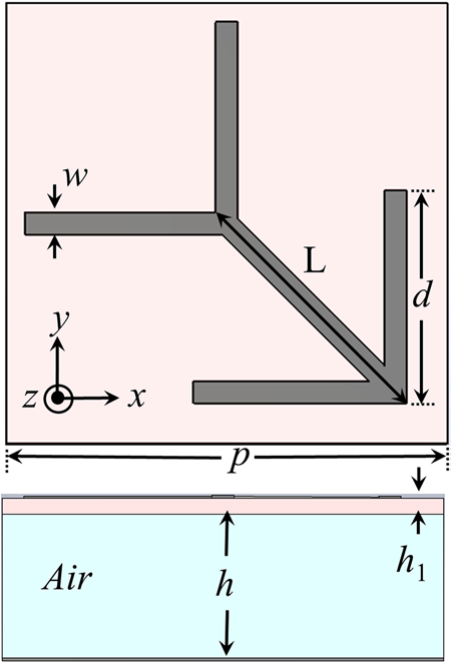
Top and side view of the double-heads arrow PCM unit cell.

The proposed unit cell is composed of two stacked layers, commercially low cost available FR-4 substrate and air as shown in Fig. 1. The double-heads arrow is printed on top of FR-4 substrate with ε_r_ = 4.4 and tan δ = 0.02 where its bottom copper layer is removed, completely. This thin FR-4 layer is spaced by an air gap from the ground copper sheet, also. The arrow is aligned diagonally to ensure the polarization insensitive behaviour of the unit cell, also. The unit cell simulation is performed by using well known commercial full wave simulation software, CST Microwave Studio. The periodic boundary conditions are considered at both *x* and *y* directions where the unit cell is excited by Floquet port to evaluate its frequency dependent response in infinite periodic structure. Figure 1 shows the geometry of the proposed double-heads arrow unit cell where the initial cell dimensions are considered as *L* = 3.4 mm, *p* = 6 mm, *d* = 2.9 mm, *w* = 0.3 mm, *h* = 2.25 and *h*_1_ = 0.25 mm. These initial dimensions are optimized to achieve the best performance in the next section.

### Polarization conversion mechanism

**Figure 2:**
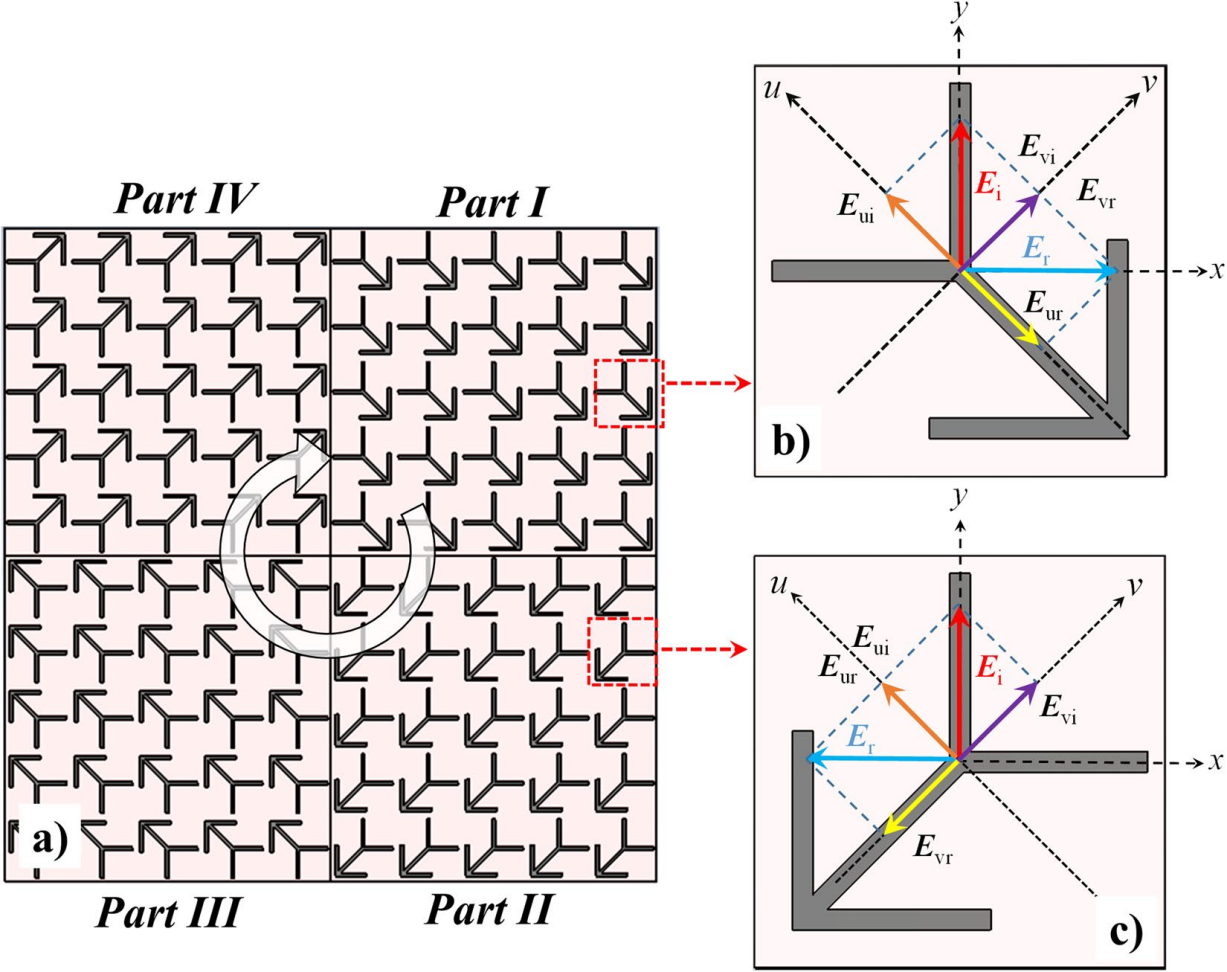
(**a**) Schematic diagram of the proposed PCM unit cell composed of the double-heads arrow unit cell (part I) with its 90° (part II), 180° (part III), and 270° (part IV) rotated ones (**b**) the decomposition of the incident electric vectors and its reflection from the PCM unit cell and (**c**) its 90° rotated one.

The polarization conversion concept of the proposed PCM unit cell is depicted in Fig. 2(b), schematically. The *u*- and *v*-axes are used to mark the unit cell anisotropic axes where *u* is the diagonal symmetrical axis as shown in the figure. The proposed double-heads arrow can be considered as combination of V-shaped resonators and cut wire resonators. The V-shaped resonators support symmetric and anti-symmetric modes which are excited by electric-field components along u and v axes, respectively^24^. While the current distribution in each arm of V-shaped resonator approximates as an antenna with first order resonance at *d* = *λ*_eff_/2 (λ_eff_ : effective wavelength) in symmetric mode, it approximates one half of an antenna with length of 2*d* in the anti-symmetric mode. The electric-field component along the *u*-axis supports multi-order dipolar resonances, also. Therefore, the combined structure has multiple plasmon resonances which are excited by electric components along *u*- and *v*-axes.

The incident planar wave with linear polarization at *y*-direction can be decomposed in *u* and *v* directions, ***E***_ui_ and ***E***_vi_. The proposed unit cell behaves as PEC in the symmetric mode which makes ***E***_ui_ and ***E***_ur_ are out-phase. In the anti-symmetric mode, the structure behaves as high impedance surface which resulting in-phase between ***E***_vi_ and ***E***_vr_. Therefore, the *y*-polarized incident wave ***E***_i_ is rotated 90° by the unit cell and converts to *x*-polarized one. This mechanism works in similar way for the mirror of this unit cell as shown in Fig. 2(c) but ***E***_i_ is rotated and directed along −*x*.

**Figure 3:**
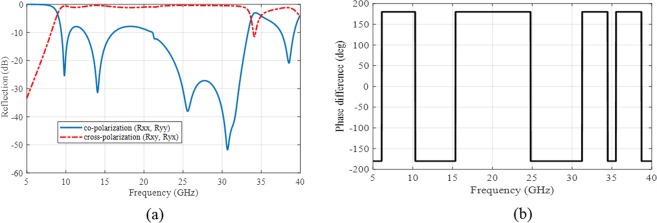
(**a**) The co- and cross-polarization reflections of the infinite periodic unit or its mirror (**b**) the cross-polarized reflection phase difference between the infinite periodic unit cell and its mirror.

To better interpret of this PCM unit cell, the co- and cross-polarization reflections of the infinite periodic unit cell or its mirror are plotted in Fig. 3(a) for normal incident wave where ${R}_{{\rm{xx}}}=\frac{|{{\bf{E}}}_{xr}|}{|{{\bf{E}}}_{xi}|}$ and ${R}_{{\rm{yx}}}=\frac{|{{\bf{E}}}_{yr}|}{|{{\bf{E}}}_{xi}|}$ represent the co- and cross polarization reflection ratios for *x*-polarized incidence wave, respectively. In similar manner, ${R}_{{\rm{yy}}}=\frac{|{{\bf{E}}}_{yr}|}{|{{\bf{E}}}_{yi}|}$ and ${R}_{{\rm{xy}}}=\frac{|{{\bf{E}}}_{xr}|}{|{{\bf{E}}}_{yi}|}$ are co- and cross polarization reflection ratios for *y*-polarized incidence wave, respectively. Notice that there are same results for *x*- and *y*-polarized incidences due to the unit cell symmetry. As it can be seen in Fig. 3, the proposed unit cell convert the incident wave polarization to its cross one from 10 GHz to 35 GHz with ${R}_{{\rm{xy}}}\,or\,{R}_{{\rm{yx}}}\approx 0\,dB$. The polarization conversion of the unit cell depicts different peaks at 10, 14, 24.77, 30 and 34.37 GHz which represent the near ideally conversion behaviour of the unit cell. Moreover, the cross-polarized reflection phase difference between the infinite periodic unit cell and its mirror (Fig. 2(c)) is plotted in Fig. 3(b) where depicts out-phase difference at the whole of bandwidth. This 180° phase difference between the proposed PCM unit cell and its mirror can be used for RCS reduction as discussed in the next section.

### RCS reduction with PCM

As discussed in the previous section, the cross polarized reflection of the PCM unit cell is almost same as its mirror but out-phase. This fact can be used to achieve the phase cancellation and RCS reduction, consequently. In more details, the mirrored PCM unit cells around *x*- and *y*- axes meet the out-phase cancellation requirement for both *x*- and *y*-polarizations. This idea is performed in the proposed RCS metasurface shown in Fig. 2(a) which includes four PCM tiles composed of the proposed unit cell (part I), its 90° (part II), 180° (part III) and 270° (part IV) rotated ones.

The polarization conversion efficiency of the proposed unit cell can be investigated by polarization-conversion ratio (PCR) for *y*-to-*x* and *x-to-y* polarizations as below which are similar due to the proposed structure symmetry

1 $${\rm{PCR}}=\frac{{{R}^{2}}_{xy}}{{{R}^{2}}_{xy}+{{R}^{2}}_{yy}}=\frac{{{R}^{2}}_{yx}}{{{R}^{2}}_{yx}+{{R}^{2}}_{xx}}$$

Since the incident wave completely reflects from the PEC bottom layer of the proposed structure, the denominator of (1) is ${{R}^{2}}_{xy}+{{R}^{2}}_{yy}={{R}^{2}}_{yx}+{{R}^{2}}_{xx}=1$ and therefore $PCR={{R}^{2}}_{xy}={{R}^{2}}_{yx}$. For simplicity, the unit cell is considered lossless, also. This means that the unit cell has good polarization conversion efficiency when *R*^2^_*xy*_ (or *R*^2^_*yx*_) has high value.

The RCS reduction can be related to the polarization conversion ratio. In more details, the proposed configuration of the PCM unit cell with its mirrors can cancel their reflected waves and redirect the energy away from the normal incidence where PCR has high enough value which results in RCS reduction. Based on (1), the high PCR in the proposed structure means high value for *R*_*yx*_, *R*_*xy*_ (e.g. 0 dB) and low value for *R*_*yy*_, *R*_*xx*_ (e.g. −10 dB). Therefore, the RCS reduction bandwidth of this mechanism can be predict form the PCR of the structure. In more details, the RCS reduction bandwidth of the surface can be predicted from the bandwidth of unit cell where *R*_xy_ (or *R*_yx_) has high enough value. Notice that the proposed PCM has good PCR value from 10 to 35 GHz.

## Design and Simulation

**Figure 4:**
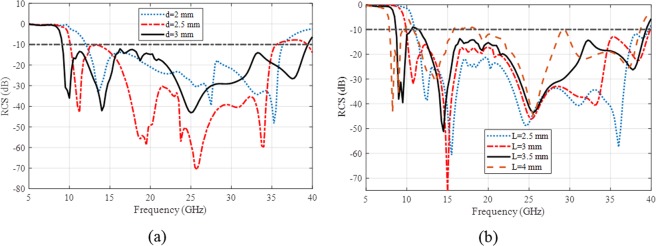
The effect of two important design parameters of the unit cell, *L* and *d* in the RCS reduction (**a**) different *d* with *L* = 3.5 mm (**b**) different *L* with *d* = 3 mm (*w* = 0.3 mm in both figures).

A 3 × 3 array of the proposed PCM tiles with its mirrors similar to Fig. 2(a) are designed and simulated to verify the proposed hypothesis. The scattering filed of this surface for normally incident wave is evaluated versus frequency which is normalized respect to a PEC ground with the same size. Figure 4 depicts the effect of two important parameters of the unit cell design, *L* and *d* in the RCS reduction where *d* = 3 mm in Fig. 4(a), *L* = 3.5 mm in Fig. 4(b) and *w* = 0.3 mm in both cases. As it can be seen, the most 10-dB RCS reduction bandwidth is achieved by *d* = 3 mm where *L* should be set between 3.5 and 4 mm. The simulation results prove that L = 3.82 mm is the optimum value where RCS is reduced more than 10-dB in the most wide bandwidth, 9 GHz to 40 GHz.

**Figure 5:**
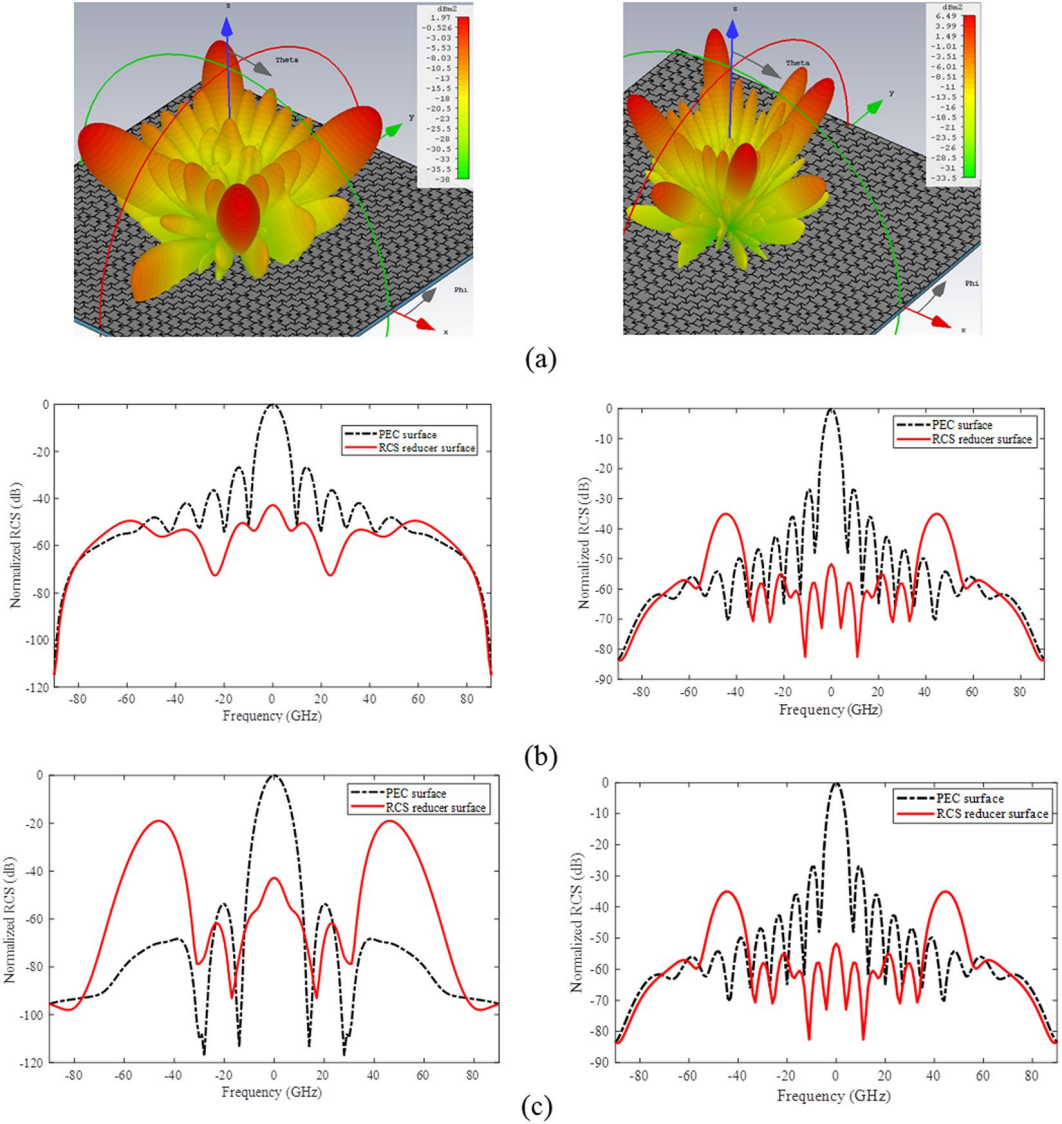
(**a**) 3D RCS pattern of the metasurface (**b**) RCS in the principal plane (φ = 0°) at 9.75 GHz (right) and 14.35 GHz (left) (**c**) RCS in the diagonal plane (φ = 45°) at 9.75 GHz (right) and 14.35 GH (left).

The 3D scattered radiation patterns of the proposed metasurface for normal *y*-polarized incident angle are shown in Fig. 5 at two representative frequencies, 9.75 GHz and 14.35 GHz which are the nulls of RCS reduction versus frequency in Fig. 4. The high ability of the surface to redirect the incident wave and reduce the RCS is clear in these figures. The reflected fields are redirected into four main lobes at diagonal planes (φ = 45°, 135°, 225°, 315°) at elevation of θ = 46° and θ = 28.5° instead of single main lobe reflected from a PEC surface. The RCS reduction of the proposed metasurface at the principal (φ = 0°) and diagonal (φ = 45°) planes are more than 42.81 dB and 19 dB, respectively at 9.75 GHz and more than 35 dB and 29 dB at 14.35 GHz compared with the maximum RCS of the metallic plate at these frequencies as shown in Fig. 5(b,c).

**Figure 6:**
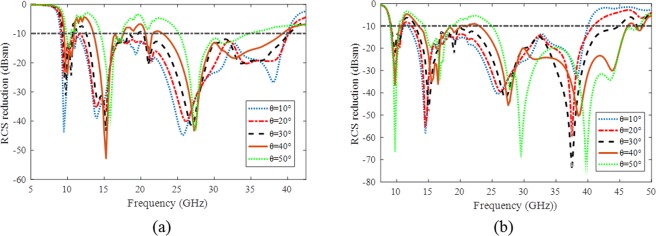
The RCS reduction of the proposed metasurface for different incident angles (**a**) TM- polarized incident waves (**b**) TE- polarized incident waves.

Since the proposed structure has mirrored arrow PCM unit cells at both *x* and *y* axes, its ability for RCS reduction is good for both TM- and TE- polarized incident waves. The performances of the surface for different incident angles are demonstrated in Fig. 6 at both TM and TE polarized oblique incident waves up to 50°. The fractional bandwidth of the surface in different polarizations and incident angles are listed in Table 1. As it can be seen, the 10-dB RCS reduction of the proposed metasurface is more than 63% for TE-polarization and more than 32% for TM-polarized ones up to 50° incident angles.

**Table 1:** The 10-dB RCS reduction bandwidths for different incident angles at both TM and TE polarizations.

Incident angle (*θ*)	TM- polarization %	TE- polarization %
10°	126.3	109.2
20°	125.35	107.7
30°	65.8	110.9
40°	52.6	67.5
50°	32.6	63.6

## Fabrication and Experimental Verification

**Figure 7:**
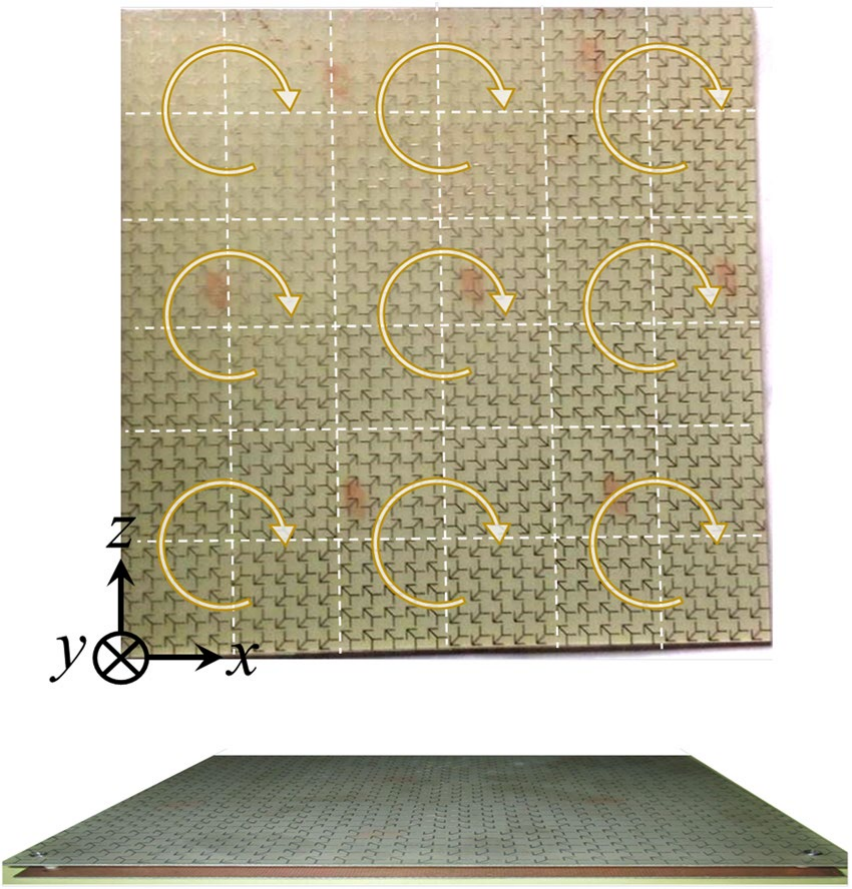
The top and side view of the fabricated PCM surface.

Figure 7 depicts the fabricated PCM RCS reducer surface which is composed of 3×3 tiles where each tiles has four sub-tiles composed of the proposed unit cell, its 90°, 180° and 270° rotated ones as marked in the figure by white dashed lines. The proposed PCM RCS reducer surface with overall dimensions 200 mm × 200 mm composed of an ultra thin low cost commercially available FR-4 substrate placed on top of a ground plane. The arrow PCM unit cells print on top layer of the FR-4 substrate with h_1_ = 0.25 mm thickness while the bottom copper of the substrate is removed, completely. This layer is spaced by an air gap from the ground copper sheet with small Teflon spacers as shown in Fig. 7, also. Therefore, the PCM unit cells have two stacked substrates, FR-4 and air with different thicknesses.

**Figure 8:**
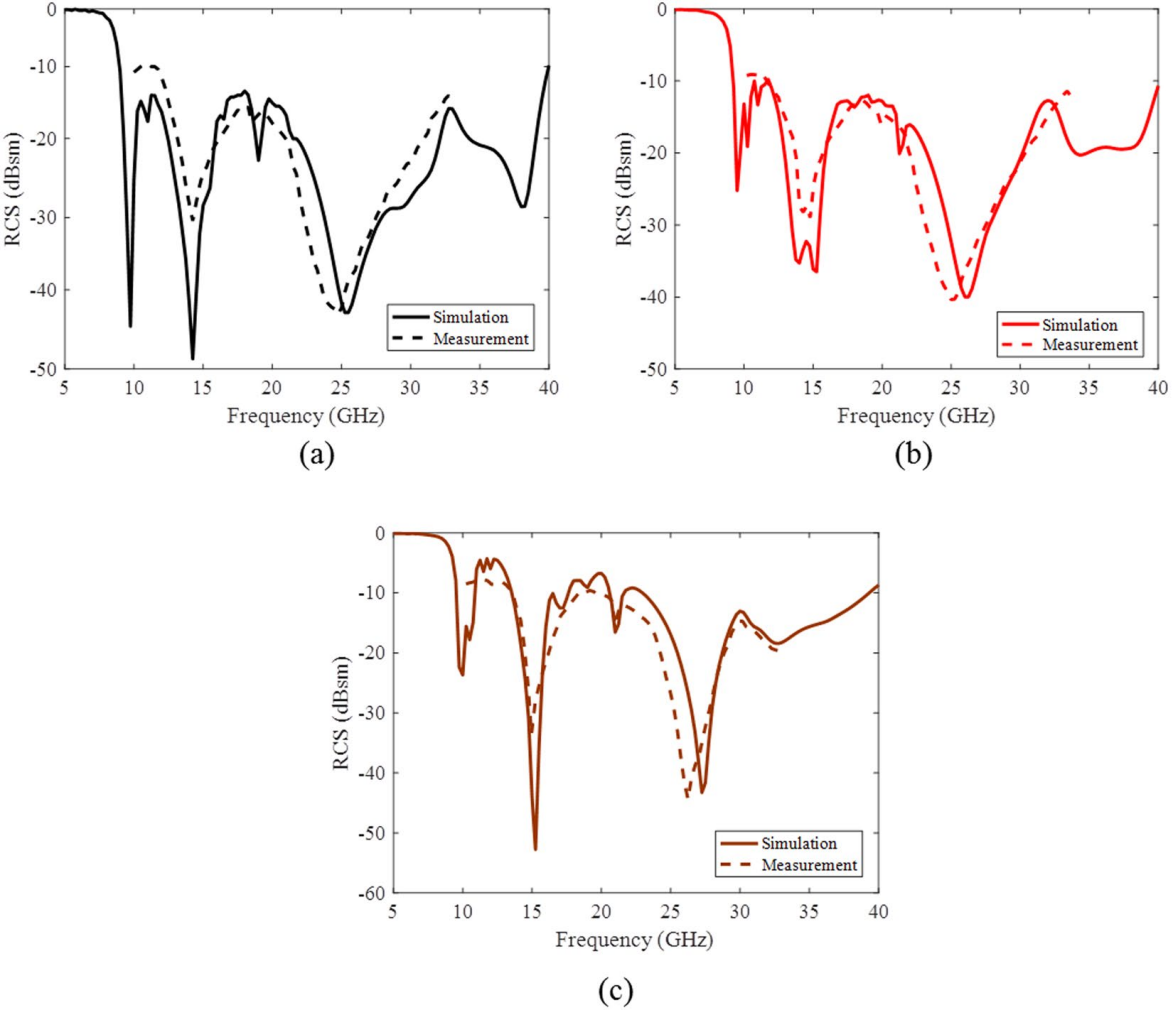
The monostatic RCS reduction of the proposed PCM surface with TM- polarization at different incident angles (**a**) normal incidence (**b**) 20° incident angle (**c**) 40° incident angle.

A simple set-up for RCS measurement of the proposed metasurface composed of two emitting standard antennas operating from 10 to 30 GHz is used to verify the numerical results. These standard antennas are used as transmitting and receiving sources in the front of the metasurface in anechoic chamber. The measurement results are normalized respect to an equal size PEC surface response. Due to the limitations of our test equipments, we only measured the RCS of the metasurface from 10 to 30.0 GHz. Moreover RCS reduction of the surface was measured for 20° and 40° TM-polarized oblique incident waves with similar setup except that the surface was tilted 20° and 40°, respectively. The measurement results are compared with the simulation ones in Fig. 8 which show very good agreement.

Table 2 compares the performance of the proposed metasurface with state of the art references. It can be seen that RCS reduction bandwidth of this structure is significantly better than the other works. It should be mentioned that the proposed metasurface is implemented on low cost ultra thin and light weight commercially available FR-4 substrate which facilitate it applications.

**Table 2:** The performance comparison of the proposed metasurface with the state of the art references.

Structure	Thickness (mm)	10-dB Bandwidth for normal incident (%)/Frequency range (GHz)	Substrate
^4^	2.28	85/9.4–23.28	RO4003
^5^	1.27	42/14.8–22.7	RO3010
^6^	6.80	95/3.8–10.7	RO4003 & PTFE
^8^	6.35	91/3.75–10	RT/duroid-5880
^11^	2.52	108/13.6–45.5	FR-4
^12^	2.52	109/13.1–44.5	FR-4
^17^	1.5	84.7/17–42	F4B-2
This paper	2.52	126.5/9–40	FR-4

## Conclusion

A new metasurface was designed to achieve ultra wideband RCS reduction by using polarization conversion technique. The simple proposed metasurface composed of double-heads arrow unit cell to obtain destructive interference cancellation between its reflected waves and its 90°, 180° and 270° rotated ones. The fabricated board depicted wideband RCS reduction larger than 10-dB from 9–40 GHz (126.5%) for normal incidence. The low profile, low cost, light weight and ultra wideband RCS reduction specifications of the proposed metasurface verify its good capability and ability compared with state of the art references.
